# The Study of Enhanced High-Intensity Focused Ultrasound Therapy by Sonodynamic N_2_O Microbubbles

**DOI:** 10.1186/s11671-019-3219-0

**Published:** 2019-12-16

**Authors:** Xiaowen Zhong, Mei Zhang, Zedan Tian, Qi Wang, Zhigang Wang

**Affiliations:** 1 The Department of Anesthesiology, The Second Affiliated Hospital of Chongqing Medical University, No. 74 Linjiang Road, Yuzhong District, Chongqing, 400010 People’s Republic of China; 2 Institute of Ultrasonic Engineering in Medical, Chongqing Medical University, Chongqing, 400010 China; 3 Chongqing Key Laboratory of Ultrasound Molecular Imaging, Second Affiliated Hospital of Chongqing Medical University, Chongqing, 400010 People’s Republic of China

**Keywords:** Nitrous oxide, Microbubble, Sonodynamic effect, HIFU, Ablation

## Abstract

High-intensity focused ultrasound (HIFU) is a representative non-invasive method of cancer therapy, but its low therapeutic efficacy and risk of damage to surrounding normal tissue hinder its further clinical development and application. Sonodynamic therapy (SDT) kills tumor cells through reactive oxygen molecules produced by sonosensitizers during ultrasound treatment. SDT can enhance HIFU efficacy like microbubbles. In this work, we developed nanoscale N_2_O microbubbles (N_2_O-mbs) by an improved mechanical oscillation method. These microbubbles showed good biocompatibility and tumor cell binding. The sonosensitivity of the N_2_O-mbs was detected both extracellularly and intracellularly through the detection of reactive oxygen species generation. The toxic effects of these sonodynamic microbubbles on tumor cells and the synergistic effect on HIFU treatment were evaluated. Significant apoptosis was caused by reactive oxygen species produced by N_2_O-mbs under ultrasound irradiation. N_2_O-mbs combined with HIFU increased tumor cell necrosis and apoptosis in vitro and the coagulative necrotic volume and echo intensity in the bovine liver target area ex vivo. These sonodynamic microbubbles have been also demonstrated to efficiently inhibit tumor growth in vivo. N_2_O-mbs have a significant impact on the treatment and ablation effect of HIFU due to the advantages of microbubble and extraordinary sonosensitivity. This finding suggests that N_2_O-mbs may be a novel auxiliary agent for ultrasound that can be used to promote HIFU tumor thermal ablation.

## Introduction

Malignant tumors are one of the major diseases that threaten human life and health [1]. High-intensity focused ultrasound (HIFU) is a new technique for the non-invasive local ablation of tumors. HIFU has been successfully used in the treatment of benign and malignant solid tumors in the liver, kidney, pancreas, prostate, breast, bone and uterus [2, 3]. However, the inherent limitations of HIFU, such as the decay of ultrasonic energy during treatment, the weakening of energy accumulation in the target area and the damage of non-target healthy tissue reduce its therapeutic effect [4, 5]. The focal field formed by HIFU irradiation is small, usually in millimeter magnitude. It takes a long time to ablate large tumors. The extension of HIFU irradiation time will increase body temperature, even reaching 39.2 °C. As the depth of the tumor increases in the body, the energy of HIFU ablation also needs to increase [6]. These increase the risk of physical damage to the patient and reduce the efficiency and safety of HIFU treatment. Therefore, researchers have committed to finding ways to increase the therapeutic efficiency of HIFU in recent years [7–9]. Microbubbles could synergistically improve HIFU therapeutic efficacy [7, 10]. The gas in the microbubble belongs to the high acoustic impedance material. The microbubbles increase the scattering and reflection of the ultrasonic wave in the target area when the HIFU irradiates the area of tumor tissue [11, 12]. Microbubbles increase the temperature of the tumor target area and enhance the thermal effect of HIFU treatment by increasing the accumulation of HIFU energy in the target area [13]. In addition, microbubbles as an exogenous cavitation nucleus also reduce the tissue cavitation threshold and enhance the cavitation effect of HIFU as it passes through the blood circulation into the tumor tissue [14]. In recent years, studies have indicated that sonodynamic therapy (SDT) can synergistically enhance HIFU treatment efficacy [15, 16]. The interactions between the sonosensitizers and ultrasound produce reactive oxygen species (ROS), including singlet oxygen and hydroxyl radicals, which kill tumor cells and inhibit tumor growth via apoptosis and necrosis [17]. SDT has made great progress in the non-invasive tumor treatment filed in recent years. It enables the reduction of drug dose and HIFU irradiation power compared to those of conventional monotherapies. In order to make full use of the advantages of the two, this study combined the microbubbles and sonodynamic effect to further improve the efficacy of HIFU. Sonosensitizers are an important part of SDT [18, 19]. Most of the currently studied traditional sonosensitizers are from photosensitizers. N_2_O is a photosensitizer [20, 21], that produce toxic substances under ultraviolet light irradiation condition. In this study, the sonosensitivity of N_2_O was also initially explored. We previously found that application of N_2_O inhalation anesthesia in HIFU operation aggravated injury degree of tissue, including the increased thickness of abdominal and skin congestion [22]. The combined utilization of N_2_O and HIFU played a synergistic effect on tissue injury. But, what biological effect N_2_O has in HIFU treatment is unclear. N_2_O is a relatively stable small molecular gas with no biotransformation and no tissue specificity in cells. It can be widely used in tissue and has no toxic effect on the body [23, 24]. So, it is an attractive prospect as a sonosensitizer. In this study, nanoscale microbubbles (N_2_O-mbs) were prepared using lipid-wrapped N_2_O and C_3_F_8_. The dosage of N_2_O was reduced and its range of action in vivo was also effectively limited to the area of tumor tissue. The sonodynamic effect of N_2_O and its synergistic effect on HIFU efficacy were examined.

## Materials and Methods

### Materials

1,2-Dihexadecanoyl-rac-glycero-3-phosphocholine (DPPC) and 1,2-distearoyl-sn-glycero-3-phosphoethanolamine-*N*-[methoxy(polyethylene glycol)-2000] (DSPE-mPEG2000) were purchased from Avanti Polar Lipids (Alabaster, AL, USA). Glycerine and agarose were obtained from Sigma Aldrich (St. Louis, MO, USA). N_2_O and C_3_F_8_ were purchased from Chongqing Ruixin Gas Co., Ltd (China). 4′,6-Diamidino-2-phenylindole (DAPI), 1,1′-dioctadecyl-3,3,3′,3′-tetramethylindocarbocyanine perchlorate (DiI), 2′,7′-dichlorofluorescin diacetate (DCFH-DA), 3-amino,4-aminomethyl-2′,7′-difluorescein, diacetate (DAF-FM DA) and CCK-8 were purchased from Beyotime Biotechnology (Chongqing, China). Calcein-AM (CAM) and propidium iodide (PI) were obtained from Santa Cruz Biotechnology (TX, USA). Annexin V-FITC/PI were obtained from Nanjing Keygen Biotech (Nanjing, China). Singlet oxygen sensor green (SOSG) was purchased from Invitrogen (NY, USA).

### Preparation of N_2_O-mbs and C_3_F_8_-mbs

N_2_O-mbs were prepared by the rotating film combined with mechanical oscillation method. We took DPPC and DSPE-PEG2000 as shell materials and N_2_O as the core. Briefly, 5 mg DPPC, 2 mg DSPE-PEG2000 and 10 mL chloroform were simultaneously poured into a round-bottom flask, mixed and fully dissolved by ultrasonic waves. Then, the lipid films were formed by rotary evaporator (60 min, 55 °C, 100 r/min). Next, 2 mL of 10% glycerol was added to hydrate the lipid films and prepare a suspension. 500 μL of which was added to a capsule tube. The capsule tube was capped with a rubber cap. The air in the tube was replaced with N_2_O and C_3_F_8_ by a gas displacement device. Then, the suspension was mechanically oscillated by a silver–mercury capsule mixer (YJT-2, Shanghai Medical Device Co., Ltd., China) for 150 s. Finally, the resulting suspension was subjected to differential centrifugation to obtain target N_2_O-mbs, and was centrifuged at 300 rpm for 5 min, and then centrifuged at 800 rpm for 5 min. N_2_O-mbs were resuspended in PBS and then stored at 4 °C for further use.

The C_3_F_8_-mbs were fabricated using the same mechanical oscillation method, as described previously [25]. DiI-labeled N_2_O-mbs can be prepared by adding DiI during spin film formation. It is worth mentioning that N_2_O has a low molecular weight and is unstable. The N_2_O in the microbubble easily overflows. Therefore, the half-life of the microbubble is reduced. C_3_F_8_ is a macromolecular inert gas that is widely used in the study of ultrasonic microbubble contrast agents. C_3_F_8_ can increase the stability of the microbubbles. Therefore, in this study, N_2_O and C_3_F_8_ (2:1) were mixed as the N_2_O-mbs core to increase their stability.

### Fabrication and Performance Detection of N_2_O-mbs

The morphology and size distribution of N_2_O-mbs were observed under bright-field optical microscopy. The concentrations of N_2_O-mbs and C_3_F_8_-mbs were calculated by globulimeter according to the calculation guidelines. The average particle size and zeta potential of the N_2_O-mbs were determined by a dynamic laser light scattering system (Malvern Instruments, Malvern, UK). The structure and morphology of the N_2_O-mbs were characterized by transmission electron microscopy (TEM, Hitachi H-7600, Hitachi Ltd., Tokyo, Japan). The prepared N_2_O-mbs were stored at 4 °C. In order to observe the stability of N_2_O-mbs, the morphology and concentration were recorded on the first, second and third days. At the same time, the average particle size and zeta potential of the microbubbles were determined.

### Cell Culture and Animal Model

Human breast cancer cells (MDA-MB-231; Jinxique Technology Development Co., Ltd. Chongqing, China) were passaged in the lab for fewer than 3 months after resuscitation. The cells were maintained in a 37 °C incubator with 5% CO_2_, using DMEM containing 10% fetal bovine serum, 50 μg/L streptomycin and 50 μg/L penicillin. When the cells reached 80% confluence, they were used for experiment.

All animal experiments were carried out according to the guidelines of Animal Ethics Committee of Chongqing Medical University. A certain number of female nude mice (4–6 weeks old; weight of 15–20 g) were purchased from Animal Experiment Center of Chongqing Medical University (Chongqing, China). For the establishment of solid tumor, MDA-MB-231 cells (1 × 10^6^ cells/mL) suspended in normal saline (100 μL) were injected subcutaneously into the right back flank of every mouse. All tumor-bearing mice were used for therapeutic experiments when the tumor volumes reached around 150 mm^3^.

### Intracellular Uptake and Biocompatibility Evaluation of N_2_O-mb

MDA-MB-231 cells in the growth phase were seeded at a density of approximately 1 × 10^5^ cells per confocal laser scanning microscopy (CLSM) flask for 24 h. Next, 100 μL (1 × 10^5^ bubbles/mL) of DiI-labeled N_2_O-mbs complete medium dilution solution was added to fill the confocal dish with medium. The dish was sealed with microplate sealers and stored inverted in an incubator, keeping the N_2_O-mbs in contact with the cells. After 2 h and 4 h of co-incubation, the cells were gently washed 3 times with PBS, fixed with 4% paraformaldehyde (PFA) for 20 min and stained with DAPI for 20 min. Finally, the cell-targeting behavior of N_2_O-mbs was observed by CLSM (Nikon A1, Japan).

The cytotoxicity of N_2_O-mbs was evaluated by a standard CCK-8 assay. MDA-MB-231 cells were seeded in a 96-well plate (5 × 10^4^ cells/well) for 24 h. Next, 100 μL of different concentrations (1 × 10^4^/mL, 1 × 10^5^/mL, and 1 × 10^6^/mL) of N_2_O-mb were added. After 24 h of incubation, CCK-8 solution (10 μL) was added to each well. Then, the MDA-MB-231 cells were cultured at 37 °C for another 2 h. Finally, the absorbance of each well was measured at 450 nm with a Bio-Tek microplate reader (Bio-Tek Instruments, Thermo Fisher Scientific, Winooski, VT, USA).

Ten healthy female nude mice approximately weighing 20 g were divided into two groups randomly. The N_2_O-mbs (*n* = 5) group were intravenously injected with N_2_O-mbs (200 μL, 1 × 10^5^/mL) and the control group (*n* = 5) injected with normal saline (200 μL). After 21 days, the major organs (heart, liver, spleen, lung and kidney) of all mice were excised and stained with hematoxylin–eosin (H&E).

### In Vitro SDT Efficiency of N_2_O-mbs

The method for oxygen radical detection was based on a previous report [26]. Briefly, 10 μL of SOSG (500 μM) was mixed with 2 mL of the sample solution. The treatments were grouped as follows: group C (the blank control group), PBS alone was added; N_2_O-mbs group, 100 μL of N_2_O-mbs (1 × 10^5^/mL) in 2 mL of PBS solution was added; C_3_F_8_-mbs group, 100 μL of C_3_F_8_-mbs (1 × 10^5^/mL) in 2 mL of PBS solution was added; LIFU group, PBS was irradiated by ultrasound (2 W/cm^2^; Institute of Ultrasound Imaging of Chongqing Medical Sciences, Chongqing, China) for 100 s; LIFU–N_2_O-mbs group, PBS with added N_2_O-mbs was ultrasound irradiated; LIFU–C_3_F_8_-mbs group, PBS with added C_3_F_8_-mbs was ultrasound irradiated. The experimental group treatment parameters were set as 2 W/cm^2^ and 100 s through the analysis and comparison of the experimental results and consultation of the literature [27, 28]. The ultrasound parameters employed for treatment purposes were set as follows: frequency, 650 kHz; focal length, 12.5 mm; pulse wave mode, 50% duty cycle; and pulse duration, 1 s. The generation of singlet oxygen (^1^O_2_) was determined by fluorescence spectrometry (Cary Eclipse, Agilent Technologies) by measuring the fluorescence intensity (*λ* excitation/*λ* emission = 504 nm/525 nm). The selection of the ultrasound intensity and duration was consistent with in vitro cell experiments.

After the generation of extracellular reactive oxygen species (ROS) was detected using the SOSG assay, the production of intracellular ROS was detected by a DCFH-DA-based ROS assay kit. MDA-MB-231 cells were seeded at a density of approximately 1 × 10^5^ cells per CLSM flask. Then, they were randomly distributed into six groups: the control group (without treatment), the N_2_O-mbs only group (100 μL, 1 × 10^5^ bubbles/mL), the C_3_F_8_-mbs only group (100 μL, 1 × 10^5^ bubbles/mL), the LIFU only group (2 W/cm^2^ for 100 s), the LIFU–C_3_F_8_-mbs group, the LIFU–N_2_O-mbs group. After incubation for 24 h to allow cell attachment, each group was subjected to the corresponding experimental intervention described above. Then, the same amount of DCFH-DA (30 μM) was added to the corresponding dish in each group, and the dish was incubated for 20 min in the dark. The cells were then gently rinsed 3 times with PBS to remove the DCFH probe that did not enter the cell. Finally, the generation of intracellular ROS was determined by CLSM. To further verify the SDT efficiency of N_2_O-mbs, cells were seeded in 6-well plates, cultured for 24 h and randomly distributed into six groups, as described above. Then, the corresponding treatments were performed in each group. The cells in each group were digested and prepared into cell suspensions at appropriate concentrations and placed into a CLSM dish. Finally, the cells were scanned by CLSM after co-staining with CAM and PI dyes to differentiate dead (red) and live (green) cells. Apoptosis was detected by flow cytometry and Annexin V-FITC/PI staining. After 1 h of treatment, each group of cells was centrifuged, and the cells were collected. Following Annexin V-FITC/PI co-staining, flow cytometry (Becton–Dickinson, Franklin Lakes, NJ) detection was performed.

### In Vitro Sonodynamic Effect of N_2_O-mbs Enhances HIFU Treatment Efficacy

A HIFU system (JC200, HIFU Technology Co., Ltd., Chongqing, China) composed of diagnostic ultrasound unit, a therapeutic ultrasound unit and a central processing system was used as described previously [29]. The HIFU parameters employed for treatment purposes were set as follows: working frequency, 0.94 MHz; focal length, 140 mm; diameter, 220 mm; and real-time diagnostic transducer frequency, 3.5 MH. The integrated transducers were submerged in degassed water. To evaluate the enhancement effect of N_2_O-mbs on HIFU treatment, MDA-MB-231 cells were centrifuged, 5 × 10^5^/mL, and distributed into four groups: the control group (without treatment), The HIFU only group (125 W, 5 s), the HIFU–N_2_O-mbs only group, the HIFU–C_3_F_8_-mbs only group. Next, the different respective treatments were performed for each group. The treated cells were centrifuged and washed with PBS, double stained with Annexin V-FITC/PI and harvested for flow cytometry. The nitric oxide (NO) probe DAF-FM was used to detect NO in the supernatant from each treatment group. All experiments were carried out 3 times. TEM was used to identify the ultrastructural morphology of the treated cells. After each treatment, the treated cells were immediately fixed with 2% OsO_4_, dehydrated in a graded series of alcohol, and flatly embedded in Epon 812 (Electron Microscopy Sciences, Fort Washington, PA). Ultrathin sections (100 nm) were prepared, stained with uranyl acetate and lead citrate, and examined under an electron microscope.

### Ex Vivo and In Vivo Sonodynamic Effect of N_2_O-mbs Enhances HIFU Ablation Efficacy

Fresh bovine liver tissues were purchased from local abattoir and used within 12 h after slaughter. Fresh ex vivo bovine liver with little connective tissue and less blood vessels was sliced into 10 cm × 5 cm × 5 cm section and degassed for 1 h at 37 °C. The bovine livers were divided into three groups: C_3_F_8_-mbs group, N_2_O-mbs group and PBS group. First, freshly isolated bovine liver was placed in a container containing deaerated water. Then, 200 μL (1 × 10^5^ bubbles/mL) of microbubbles in PBS was directly injected into the bovine liver in the manner shown in Fig. 6a. Under the guidance of a diagnostic ultrasound transducer, the HIFU focal point was positioned at the injection site. Then, the therapeutic transducer was used to perform 5 s of ablation at different power (100 W, 125 W, 150 W) on the bovine liver injection site. Finally, the coagulative necrotic volume of the target area in the bovine liver tissue was calculated according to the measured maximum length, width and depth (mm) by the following equation: *V* (mm^3^) = π/6 × length × width × depth. Furthermore, the grey value of the target area before and after HIFU ablation in each group was recorded on the diagnostic ultrasound images.

Twenty tumor-bearing mice were randomly divided into four groups (*n* = 5 per group), including a control group (without any treatment), a HIFU–PBS group, a HIFU–C_3_F_8_-mbs group, and a HIFU–N_2_O-mbs group. HIFU (125 W, 5 s) ablation of tumor was applied after the mice received an intravenous injection of 200 μL of C_3_F_8_-mbs group, N_2_O-mbs and PBS. The HIFU ablation procedure of solid tumors was similar to that of ex vivo isolated bovine liver. Firstly, mice were anesthetized with 1% pentobarbital (8 mL/kg). The tumor site of the mouse was inoculated into a container containing deaerated water. Under the guidance of a diagnostic ultrasound transducer, the HIFU focal point was positioned at the tumor tissue. The mice body weights and the tumor volume were recorded with a 3-day interval. Finally, all mice were killed 21 days after treatment and tumor tissues were dissected from the mice and then sent for H&E, proliferating cell nuclear antigen (PCNA) staining, terminal deoxynucleotidyl transferase dUTP nick end labeling (TUNEL).

### Statistical Analysis

All data were presented and analyzed with GraphPad Prism (version 7, GraphPad Software Inc., La Jolla, CA, USA). Data are expressed as the mean ± SD. Each experiment was repeated at least 3 times. Statistical analyses were performed by one-Way ANOVA with Bonferroni post-test (comparing all groups) or Dunnett post-test (comparing all groups to a control group). A value of *p* < 0.05 was considered statistically significant.

## Results

### Characterization of N_2_O-mbs

**Fig. 1:**
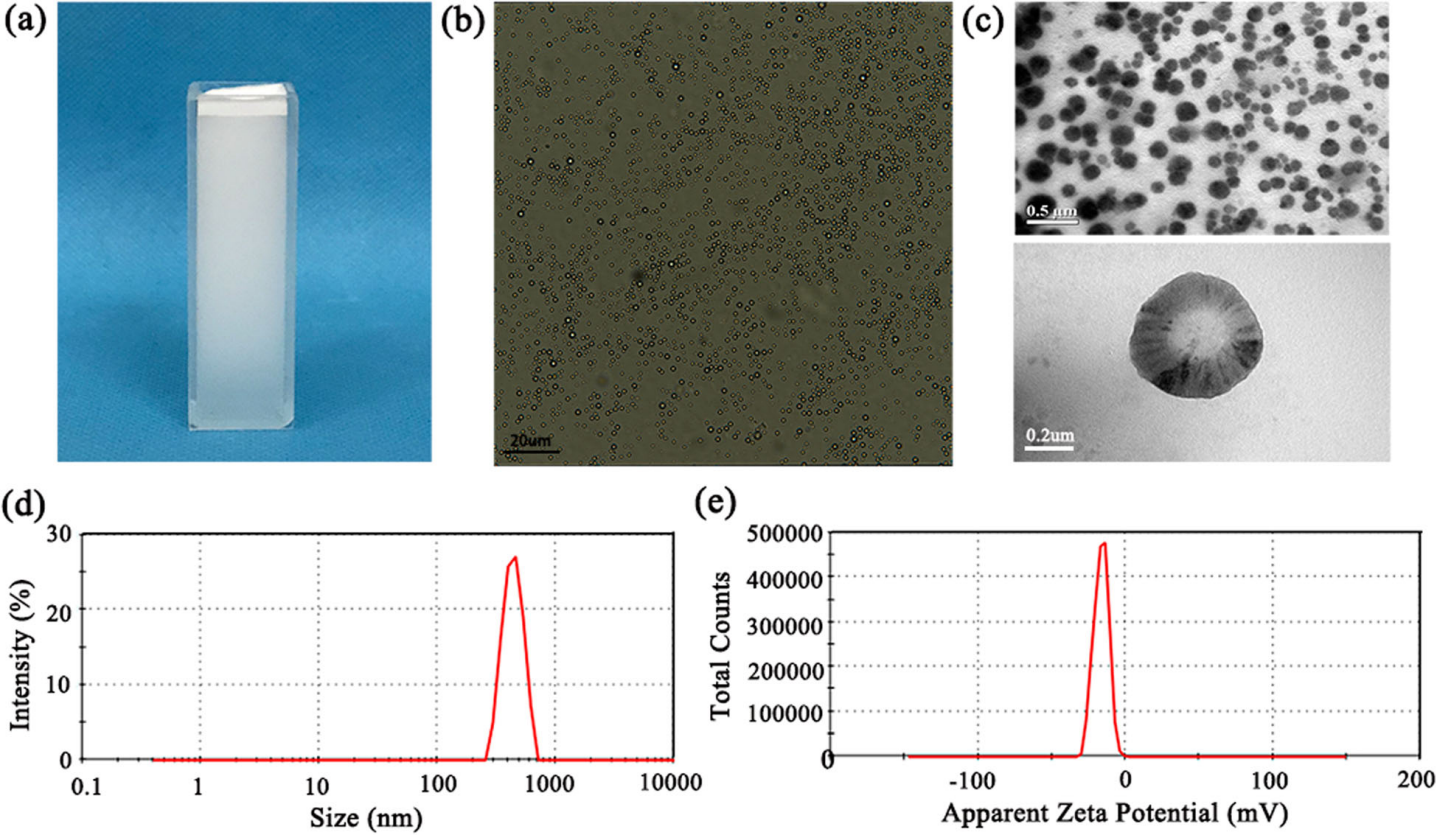
**a** Photographs of N_2_O-mbs dispersed in deionized water; **b** image of N_2_O-mbs under bright-field optical microscopy; **c** transmission electron microscope image of N_2_O-mbs; **d** size distribution of N_2_O-mbs; **e** apparent zeta potential of N_2_O-mbs

The preparation of N_2_O-mbs by the modified mechanical oscillation method results in a highly stable milky white suspension (Fig. 1a). The N_2_O-mbs did not change significantly in particle size and morphology after 72 h of storage at room temperature. Under the light microscope, N_2_O-mbs were observed with uniform size, good dispersion and high stability (Fig. 1b). The concentration of N_2_O-mbs was 5.12 ± 0.45 × 10^8^ bubbles/mL. As shown in the TEM images (Fig. 1c), the size of N_2_O-mbs was evenly distributed, and the mean size was was approximately 450 nm. The N_2_O-mb dynamic light scattering results revealed hydrodynamic diameters of 458.1 ± 17.26 nm (polydispersity index = 0.368; Fig. 1d), which was consistent with the TEM results. The potential of the N_2_O-mbs was − 16.2 ± 0.35 mV (Fig. 1e) (Additional file 1: Figure S1).

### Intracellular Uptake and Biocompatibility of N_2_O-mbs

**Fig. 2:**
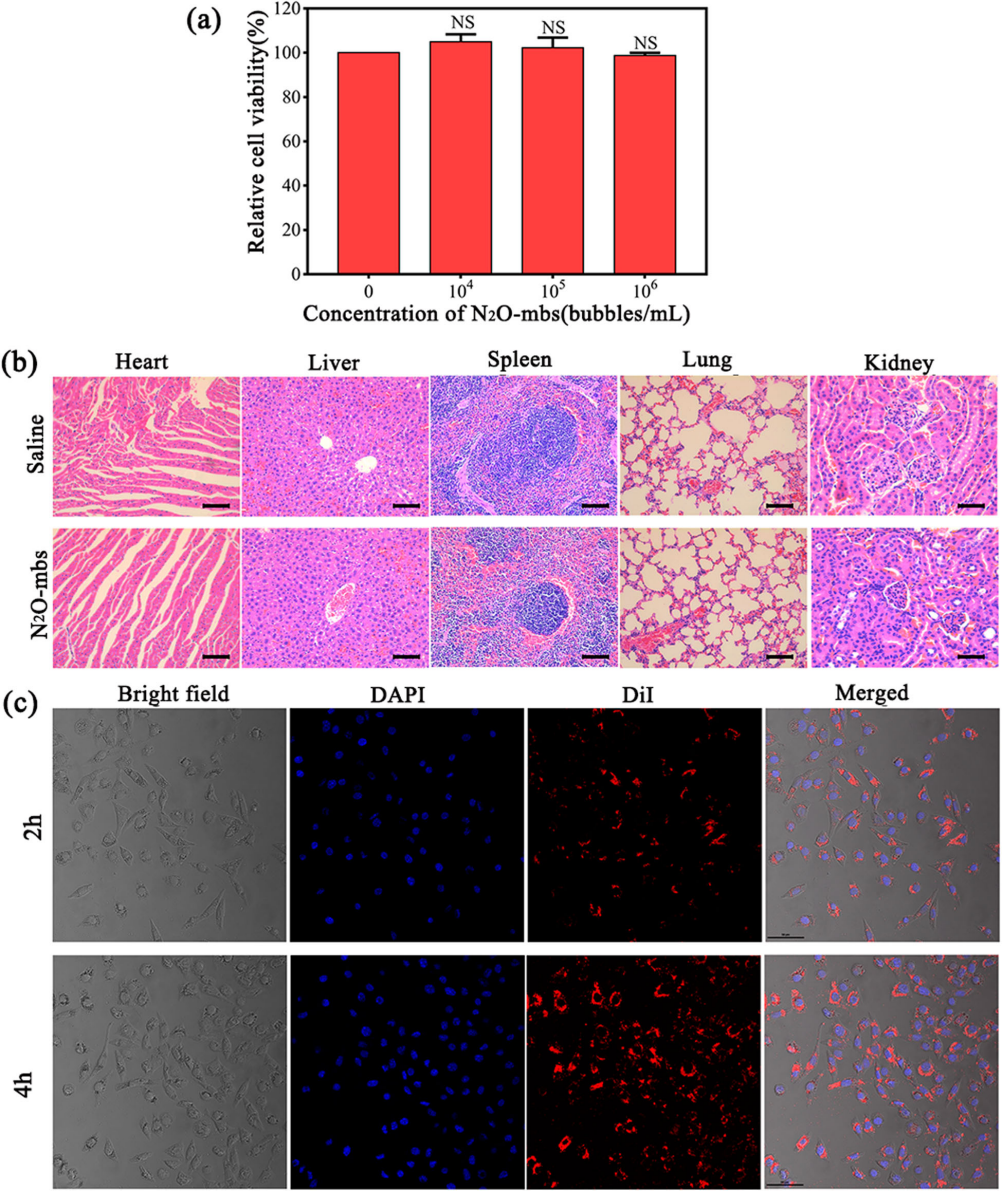
**a** Cell viability was determined by CCK-8 assay; **b** H&E staining of the major organs (heart, liver, spleen, lung and kidney) in healthy female nude mice with or without receiving the N_2_O-mbs; **c** the images from **a** to **d** show cell in bright field, cell nuclei stained by DAPI, N_2_O-mbs stained by DiI, and the merged results of the three fluorescence images; scale bar = 50 μm [*NS* no significance, compared with control group (0 bubbles/mL)]

The cytotoxicity of N_2_O-mbs against cells in vitro was investigated by the CCK-8 method. We defined the cell viability of the control group as 100%. After 24 h of incubation, the cell viability results showed no significant cytotoxicity to MDA-MB-231 cells. Even at high N_2_O-mb concentrations, the cell viability remained greater than 95% (Fig. 2a). The cytotoxicity of N_2_O-mbs in vivo on healthy nude mice was analyzed. As shown in Fig. 2b, histopathology analysis of main organs (heart, liver, spleen, lung and kidney) showed that N_2_O-mbs did not cause significant toxic damage to mice. These results indicated that N_2_O-mbs have high biocompatibility.

The intracellular uptake of N_2_O-mbs over prolonged incubation times (2 h and 4 h) was visualized by CLSM. The incubation of cells with DiI-labeled N_2_O-mbs resulted in the accumulation of a large number of N_2_O-mbs in the cancer cell membrane and cytoplasm. As shown in Fig. 2c, there was good colocalization of red fluorescent N_2_O-mbs with the blue fluorescent cells. This finding suggests that N_2_O-mbs are efficiently internalized into MDA-MB-231 cancer cells.

### Sonodynamic Effect of N_2_O-mbs In Vitro

**Fig. 3:**
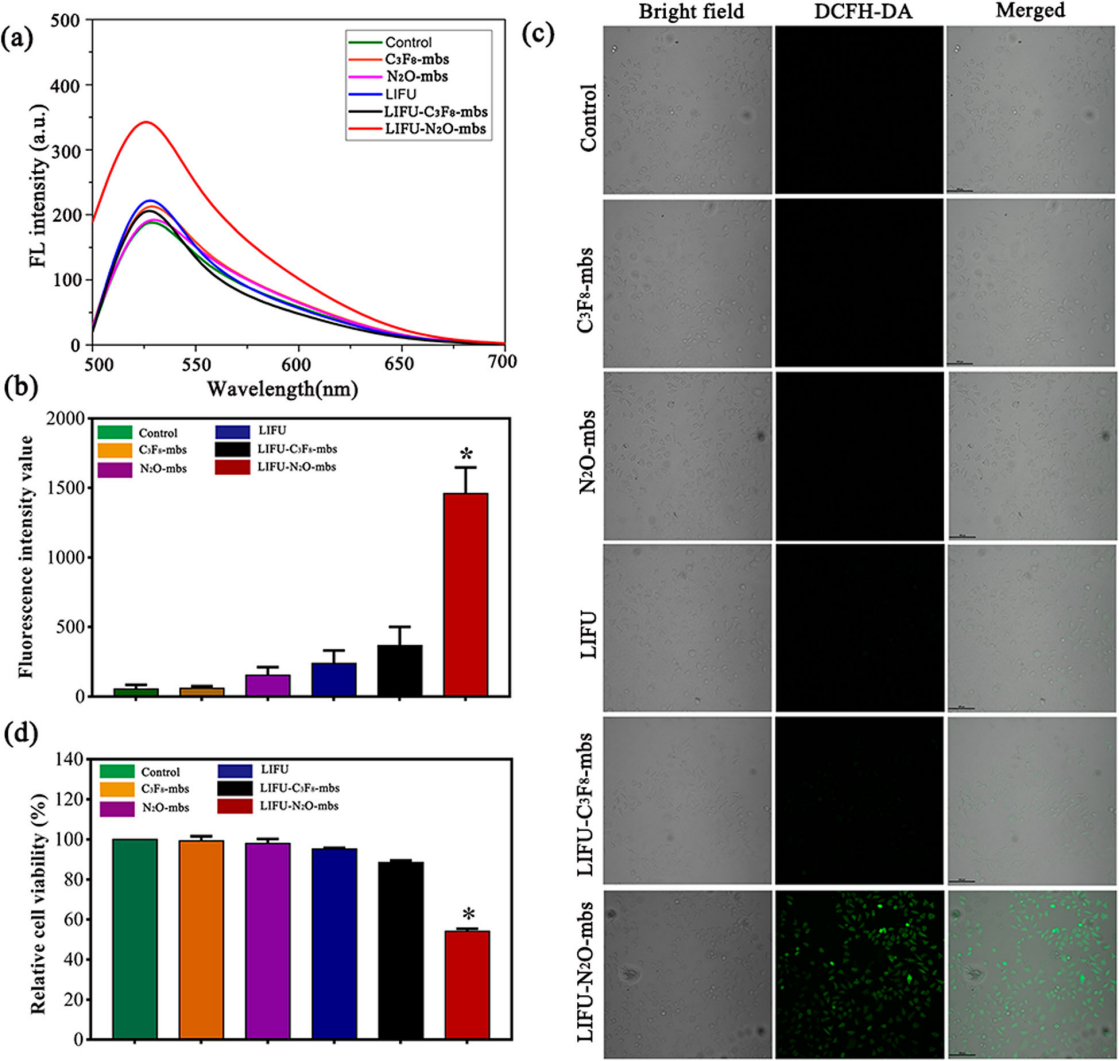
**a** Fluorescence spectrum of SOSG with N_2_O-mbs irradiated by LIFU; **b** cell viability was determined by CCK8 assay; **c** confocal images of intracellular ROS generation (green fluorescence indicates positive staining for ROS stained with DCFH-DA); **d** the average fluorescence intensity value (DCFH-DA) of each group calculated by Image J. (The data were shown as mean ± SD, *n* = 5 per group, *p < 0.05, compared with LIFU–C_3_F_8_-mbs group)

SOSG is a commonly used singlet oxygen detection probe that is highly sensitive and reliable for the detection of oxygen free radical generation. To explore the potential of N_2_O-mbs as a sonosensitizer, SOSG was used to detect ROS generation in vitro [30, 31]. SOSG combines with ^1^O_2_ to form SOSG-EP, which is characterized by an increase in fluorescence intensity. As shown in Fig. 3a, after ultrasonic irradiation of SOSG solution containing N_2_O-mbs, the fluorescence intensity value increased significantly; compared with that in the rest of the groups, the production of oxygen free radicals was significantly increased (*p* < 0.01).

**Fig. 4:**
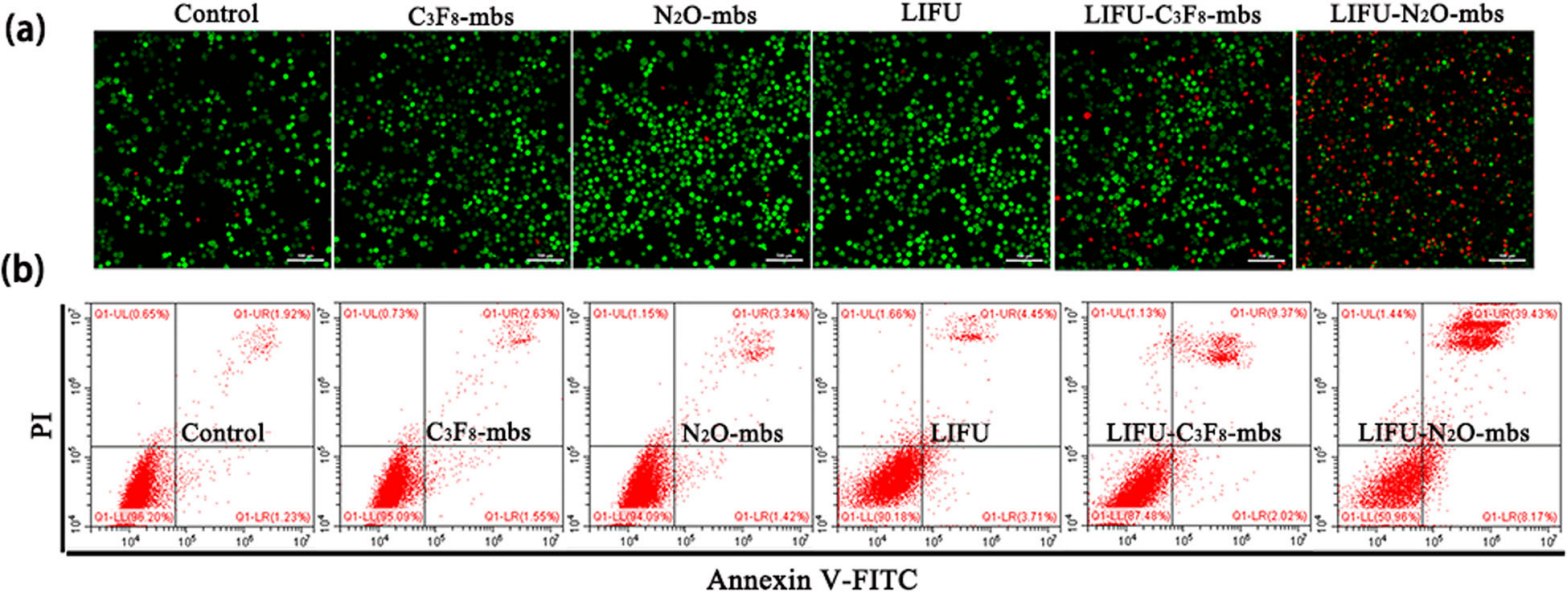
**a** Flow cytometry analysis of tumor cell apoptosis and necrosis; **b** confocal images of CAM/PI co-stained MDA-MB-231 cells after various treatments. The scale bars are 100 μm

In addition to the detection of ROS generation in aqueous solution after ultrasonic irradiation of N_2_O-mbs, a DCFH-DA ROS assay kit was used to determine the intracellular ROS production by N_2_O-mbs. As shown in Fig. 3b, the positive control group demonstrated the strongest fluorescence intensity. The next strongest green fluorescence signal appeared only in the LIFU–N_2_O-mbs group, revealing the consequential production of ROS in the simultaneous presence of N_2_O-mbs and LIFU irradiation. However, no obvious fluorescence was detected in any of the remaining groups (Fig. 3c). The cell viability of the control group was defined as 100%. The CCK-8 assay results showed approximately 46% cell death in the LIFU–N_2_O-mbs group (Fig. 3d). The cytotoxicity of N_2_O-mbs in combination with LIFU was evaluated by CLSM observation of cell death after treatment. CAM was used to stain live cells with green fluorescence. PI was used to stain dead cells with red fluorescence. As shown in Fig. 4a, there were many dead cells in the LIFU–N_2_O-mbs group. Furthermore, the flow cytometry results showed that the apoptosis rate of the MDA-MB-231 cell group was significantly higher than that of other groups (*p* < 0.05) after the combined N_2_O-mbs with LIFU treatment (Fig. 4b). The combined C_3_F_8_-mbs with LIFU treatment did not cause obvious cell death or apoptosis. The results further indicated that extensive apoptosis and necrosis occurred in response to SDT. The flow cytometry results were consistent with those of the CLSM. These results indicate that N_2_O-mbs combined with LIFU induce tumor cell apoptosis in vitro (Additional file 2: Figure S2; Additional file 3: Figure S3).

### In Vitro HIFU Synergistic Effect Assessment in Cancer Cells

**Fig. 5:**
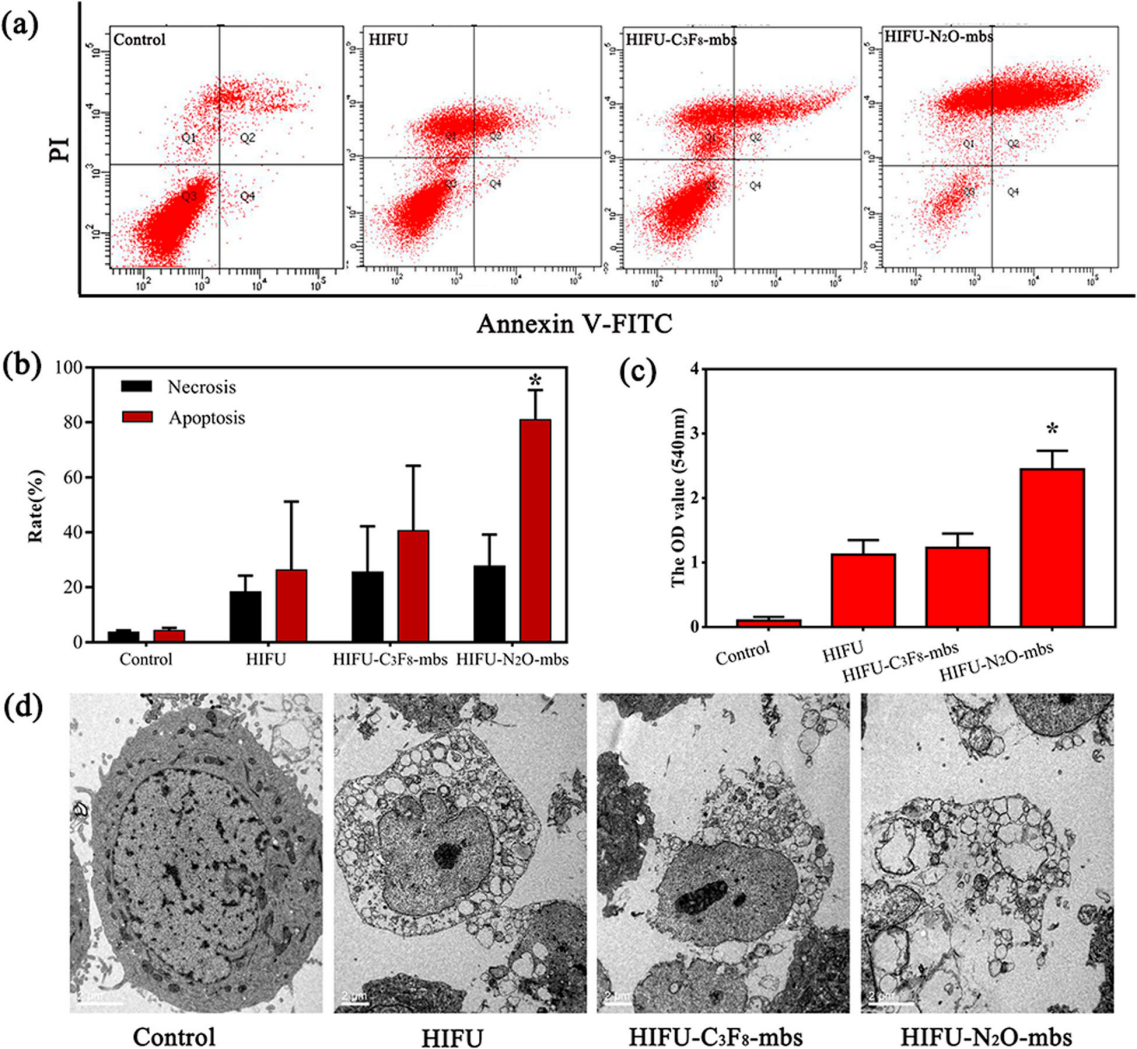
**a** Results of Annexin V-FITC/PI binding assay; **b** the average cell apoptosis rate in each group; **c** quantitative analysis of NO generation of N_2_O-mbs irradiated by HIFU; **d** transmission electron microscopy image of cells. The scale bars are 100 μm. (The data were shown as mean ± SD, *n* = 5 per group, *p < 0.05, compared with control, *HIFU*, HIFU–C_3_F_8_-mbs groups)

The flow cytometry results are shown in Fig. 5a. Both N_2_O-mbs and C_3_F_8_-mbs increased the mortality of MDA-MB-231 cells after HIFU ablation. Furthermore, compared to HIFU-C_3_F_8_-mbs, N_2_O-mbs significantly increased MDA-MB-231 apoptosis (*p* < 0.05; Fig. 5b). The results of the NO test showed that the fluorescence intensity of the supernatant of the HIFU–N_2_O-mbs group was also significantly higher than that of the remaining groups (*p* < 0.05) (Fig. 5c). Changes in the cytoplasm and organelles were observed by TEM (Fig. 5d). The cell in the control group (without any treatment) showed complete cell morphology. Large amounts of vacuolar substances were found in the cell cytoplasm of the HIFU group. But the cells still have complete cell membranes and nuclear membranes. However, both the HIFU–N_2_O-mbs group and the HIFU–C_3_F_8_-mbs group showed a loss of cell membrane integrity and complete disintegration of the cell structure. It was worth mentioning that the TEM observations were only the moment in the process of cell death and apoptosis after the treatment in each group. N_2_O-mbs as exogenous microbubbles have an obviously synergistic effect on the HIFU treatment. In the HIFU–N_2_O-mbs group, the sonodynamic effect of N_2_O-mbs could promote more cell apoptosis. The results have been validated in both flow cytometry detection and in vivo experiment (Additional file 4: Figure S4; Additional  file 5: Figure S5)

### N_2_O-mbs as a Synergist for HIFU Therapy

**Fig. 6:**
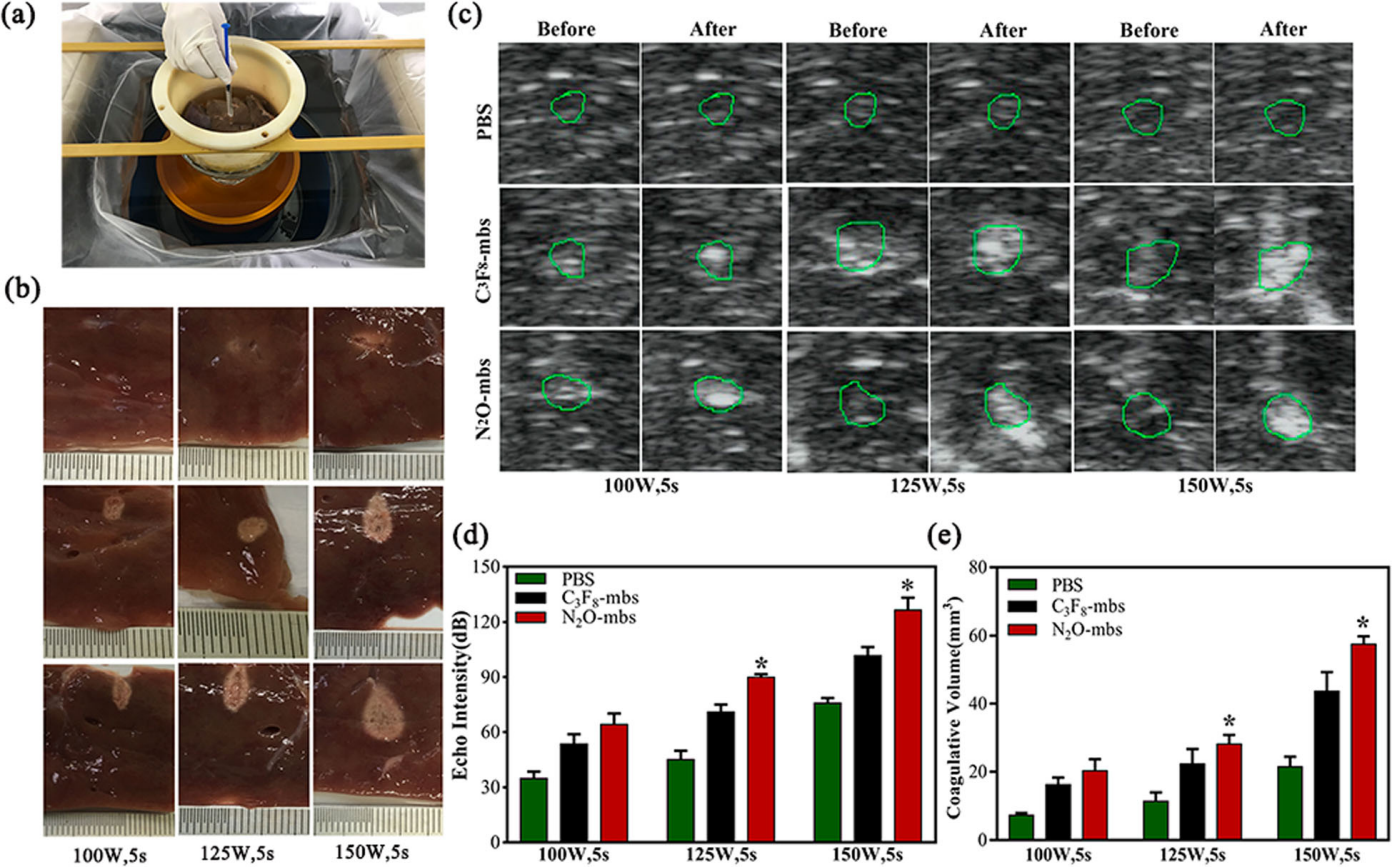
**a** Schematic illustration of ex vivo HIFU ablation on degassed bovine livers; **b** photography of the targeted area in excised bovine liver after HIFU ablation; **c** real-time ultrasound images before and after HIFU irradiation on bovine livers at 100 W, 125 W and 150 W for 5 s; **d** quantitative analysis of echo intensity; **e** quantitative analysis of coagulative necrosis volume of the targeted area in the excised bovine liver after HIFU ablation. (The data were shown as mean ± SD, *n* = 5 per group, *p < 0.05, compared with 100 W, 5 s, 125 W, 5 s groups)

After HIFU ablation of the isolated bovine liver, a grey–white coagulative necrotic region appeared in the targeted area (Fig. 6b). The average echo intensity changes in the target area before and after HIFU are shown in Fig. 6c. Compared with those of the PBS group, the target echo intensity values of the C_3_F_8_-mbs group and the N_2_O-mbs group were significantly increased after HIFU treatment (*p* < 0.05) and increased as the HIFU ablation intensity increased. The coagulative necrotic volume of the latter two groups and the echo intensity of the B-mode ultrasound images of the target area both increased as the HIFU power increased (Fig. 6d, e).

**Fig. 7:**
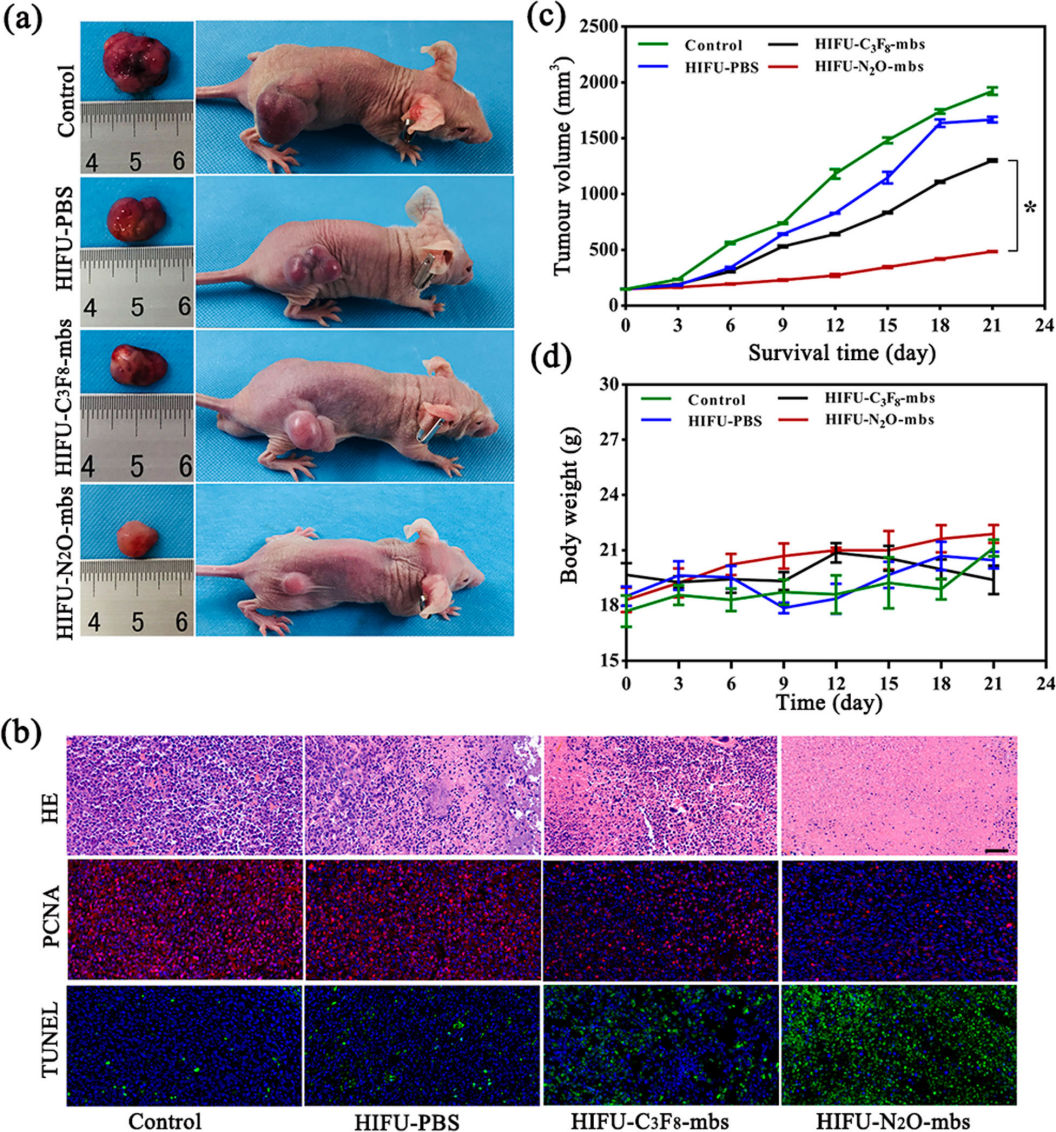
**a** Digital photos of tumor-bearing mice and ex vivo tumors 21 days after different treatments; **b** H&E, PCNA and TUNEL staining of tumor sections after various treatments; **c** the corresponding calculated tumor volume of each group after 21 days of different treatments; **d** body weight of tumor-bearing mice for each group. The scale bars in HE staining are 100 μm; the scale bars in TUNEL and PCNA staining are 50 μm. (The data were shown as mean ± SD, *n* = 5 per group, *p < 0.05, compared with HIFU-C_3_F_8_-mbs group)

The synergistic effect of N_2_O-mbs on HIFU therapy was further verified in vivo. As shown in Fig. 7a, the digital photos of mice and corresponding tumor tissue were recorded 21 days after different treatment. Tumor growth was more effectively inhibited after N_2_O-mbs combined with HIFU treatment. The synergistic effect of N_2_O-mbs on HIFU treatment was also evaluated by H&E, PCNA, and TUNEL staining (Fig. 7b), which demonstrated that N_2_O-mbs combined with HIFU irradiation could cause larger scale of tumor cell necrosis as compared with HIFU–C_3_F_8_-mbs group. The cell morphology changes exhibited in HIFU–N_2_O-mbs were more obvious than other groups, including karyopyknosis, karyorrhexis and karyolysis, indicating the substantial necrosis of cancer cells.

In addition, the tumor volume of HIFU–N_2_O-mbs exhibited a more significant inhibited tendency than HIFU–C_3_F_8_-mbs within the 21 days (Fig. 7c, d).

## Discussion

HIFU, as a new method of oncotherapy, has been clinically recognized and has achieved notable progress in the treatment of many solid tumors [32]. The thermal effect, cavitation effect, mechanical effect and acoustic chemical effect play significant roles in killing tumor cells and causing irreversible damage to the target area tissue during HIFU ablation [33]. HIFU has limited impact on surrounding normal tissues and is essentially a non-invasive tumor treatment. However, the energy carried by the ultrasound is attenuated due to increased transmission distance, absorption by the blood flow in the target area and the blockage of bones or gas in the body during the HIFU treatment, so that the energy transferred to the target area is reduced. However, prolonging the ablation time and increasing the treatment power increases the risk of normal tissue damage, such as skin burns, neurovascular damage and other complications [34]. Therefore, it is necessary to increase the efficacy of HIFU. Many studies have confirmed that both microbubbles and SDT can improve the efficacy of HIFU [15, 16, 35]. The N_2_O-loaded microbubbles prepared in this study have a smaller particle size, but similar characteristics as ultrasonic microbubble contrast agents. Studies have shown that nano/microbubbles have the same imaging capability as microbubbles in vitro, but have greater capability than microbubbles in vivo [36]. So, N_2_O-mbs not only could be used for ultrasound guidance and localization for HIFU treatment, but also could be used as exogenous microbubbles to enhance the efficacy of HIFU (Additional file 6: Figure S6). At the same time, the sonodynamic effect of N_2_O-mbs could effectively improve the efficacy of HIFU. The generation of ROS after ultrasonic excitation is one of the most important factors for sonosensitizers [16]. To verify the sonosensitivity of N_2_O-mbs, the production of ROS in both extracellular and intracellular environments was detected in this study. The consistent result of both tests showed that N_2_O-mbs can generate ROS in both aqueous solution and intracellularly. Studies have also shown that gases (N_2_ and O_2_) trapped in cavitation nuclei in solution can undergo cleavage reactions and produce N and O free radicals [37, 38], as follows:

$$ {N}_2O\to {N}_2+{O}_2\kern0.50em {O}_2\to 2\bullet O\ \ \ {N}_2\to 2N.N.+ HO.\to NO+H. NO+ HO.\to HNO $$

The cavitation effect and the advantage of microbubble packaging reduce the difficulty of the N_2_O reaction [39]. When incubated with cells, N_2_O-mbs exhibit good biocompatibility. The high selectivity, high affinity and high drug loading of nanoparticles [40], as well as the active phagocytosis by cells, promote efficient N_2_O-mbs binding to cancer cells. The nanobubbles display increased penetration, stability and ease of cell entry or aggregation around the cells [36]. In addition, the sonoporation effect of ultrasound can effectively promote the transfer of microbubbles into cells [41].

In contrast to the traditional microbubble contrast agent C_3_F_8_-mbs, N_2_O-mbs have a greater inhibitory effect on cell growth after ultrasonic irradiation, which is characterized by increased intracellular ROS, decreased cell viability, and apoptosis and necrosis. These results preliminarily suggested the sonodynamic toxic effect of N_2_O-mbs.

In the HIFU synergy experiment, the treatment parameters in the experimental group were set to 125 W for 5 s by analyzing and comparing the experimental results and consulting the literature, which validated a low HIFU power could induce sufficient ablation effect and reduce adverse effects. The results showed that N_2_O-mbs increased the necrosis and advanced apoptosis of more tumor cells. N_2_O-mbs acted as a nanoscale microbubble synergizer and its sonodynamic effect in response to HIFU may be the reason for the different experiment results. As a microbubble, N_2_O-mbs increased the deposition of HIFU energy in the target area and increased the temperature of the target area and increased the thermal effect of HIFU treatment [11]. The generation of the cavitation effect is closely related to the cavitation threshold and cavitation nucleus concentration [42]. N_2_O-mbs were cavitation nucleus that reduce the cavitation threshold of HIFU and facilitate the cavitation effect. Therefore, N_2_O-mbs effectively promoted cell necrosis. As a nanoscale microbubble synergizer, N_2_O-mbs were lysed and generate free radicals and nitrogen oxide in response to ultrasound. Studies have confirmed that ROS are important factors in apoptosis induction [43]. It is worth noting that many kinds of free radicals can cause damage to cells. In addition to the generation of oxygen radicals, N_2_O can generate other types of free radicals in cleavage reactions, such as nitrogen free radicals, which contribute to the killing of tumor cells. A small amount of nitric oxide (NO) production was detected in the cell supernatant after HIFU treatment, further indicating that the N_2_O cleavage reaction occurred. An et al. [44] reported that NO may have an anti-tumor function, the mechanism of which involves NO attacking suspected iron enzymes that cause cells to fail to grow and divide when they are trapped. In addition, unstable NO can interact with oxygen molecules to form hydroxyl radicals (HO^·^) and NO_2_. Hydroxyl radicals are extremely cytotoxic and promote tumor cell apoptosis. NO is also an endothelium-derived relaxing factor. The generation of NO may be the reason for hyperaemia and abnormal swelling in patients during HIFU operation. In an excised bovine liver experiment, we carefully compared the echo intensity of B-mode ultrasound images, pathological changes and coagulative necrotic volume of the target area after HIFU ablation in the presence of N_2_O-mbs, C_3_F_8_-mbs and PBS. All of these results were consistent. Compared with PBS, N_2_O-mbs effectively enhanced the ablation effect of HIFU. This synergistic effect was also better demonstrated in the ablation of solid tumor in vivo, manifested by tumor cell apoptosis increased, tumor volume reduced and tumor growth inhibition. This finding further indicates that N_2_O-mbs may be a novel auxiliary agent for ultrasound that can be used to promote HIFU thermal tumor ablation. The synergistic effect of N_2_O-mbs in HIFU and the synergistic mechanism provide new ideas and theoretical practice for HIFU synergist research. But, the other related mechanisms of the synergy should be further explored. The effect of tumor ablation by HIFU combined with N_2_O-mbs should be further addressed.

## Conclusion

In summary, we have developed N_2_O-mb nanoparticles. The characterization and biocompatibility of the N_2_O-mbs were determined. The sonosensitivity of N_2_O-mbs was examined by detecting the production of ROS in aqueous solution and intracellularly. Compared with C_3_F_8_-mbs, N_2_O-mbs generated ROS and promoted the apoptosis and necrosis of tumor cells, which exhibited extraordinary sonosensitivity. In the HIFU synergy experiments, the sonodynamic effects of N_2_O-mbs significantly increased the apoptosis and necrosis of tumor cells in vitro, and increased the coagulative necrotic volume and echo intensity in the ex vivo isolated bovine liver target area, and effectively inhibited tumor growth in vivo. These results suggested that N_2_O-mbs could be a new kind of sonosensitizer and a novel auxiliary agent for ultrasound therapy that can be used for the ablation of tumors by HIFU.

## Supplementary information

**Additional file 1: Figure S1.** Basic representation of C_3_F_8_-mbs. A. Photographs of C_3_F_8_-mbs dispersed in deionized water; B. Image of C_3_F_8_-mbs under bright-field optical microscopy; C. Transmission electron microscope image of C_3_F_8_-mbs; D. Size distribution of C_3_F_8_-mbs; E. zeta potential of C_3_F_8_-mbs.

**Additional file 2: Figure S2.** Determination of optimal LIFU irradiation time. (The data were shown as mean ± SD, *n* = 5 per group, *p < 0.05). To Select the optimal time for ultrasound, MDA-MB-231 cells were seeded in a 96-well plate at a density of 5000 cells per well for 24 h. And then use the strength of 2 W/cm^2^ ultrasonic processing cells, respectively for the 50 s, 100 s, 150 s and 200 s. After the indicated treatments, CCK8 solution (10 μL) was added in each well. Then, the MDA-MB-231 cells were cultured at 37 °C, 5% CO_2_ cell incubation box to save. At 3 h, 6 h, 9 h and 12 h, cell viabilities were determined by CCK-8 assay. The optical density of each well was measured at 450 nm with a Bio-Tek microplate reader. Cell viability of the control group was defined as 100%. The test results show (Figure S1) that the cells viability in 50 s and 100 s groups were more than 95% at 3 h, 6 h, 9 h and 12 h after treatment, and the difference in cell viability between the two groups is not statistically significant (*p* > 0.05). The cell viability decreased in 150 s and 200 s groups (*p* < 0.01), and the survival rate was 74% in 150 s group and 55% in 200 s group. Combined with the oxygen free radicals generated condition, 100 s was selected as the optimal time of ultrasound.

**Additional file 3: Figure S3.** Quantitative analysis of ^1^O_2_ generation of N_2_O irradiated by LIFU. (The data were shown as mean ± SD, *n* = 5 per group, *p < 0.05). In aqueous solution, the difference between the N_2_O treated with ultrasound (LIFU-N_2_O group) and the N_2_O group was not statistically significant (*p* > 0.05). Compared with the control group, fluorescence intensity increased only in the LIFU group and LIFU-N_2_O group. And the addition of N_2_O did not increase the generation of oxygen free radicals in the LIFU-N_2_O group (*p* > 0.05). In the LIFU group and LIFU-N_2_O group, a small amount of oxygen free radicals was generated, possibly only because of the stimulation of ultrasound.

**Additional file 4: Figure S4.** HIFU ablation cell suspension.

**Additional file 5: Figure S5.** HIFU intensity and time selection. (The data were shown as mean ± SD, *n* = 5 per group, *p < 0.05). The prepared MDA-MB-231 cells suspension was grouped and treated with different acoustic power (75 W, 100 W, 125 W, 150 W, 175 W, 200 W) and different time (5 s, 10 s). Cell viability was detected with CCK8 assay. After the indicated treatments, MDA-MB-231 cells were inoculated in 96-well plates, with 5 wells in each group. CCK8 solution (10 μL) was added in each well and cultured at 37 °C for another 1 h. The optical density of each well was measured at 450 nm with a Bio-Tek microplate reader. Cell viability of the control group was defined as 100%. When the time was 5 s, the cell viability rate decreased significantly when the sound power was higher than 125 W (*p* < 0.05), and the cell survival rate no longer changed with the increase of the sound power (*p* > 0.05). At the same sound power and time of 10 s, the cell survival rate of each group did not change significantly. According to the experimental conditions, 125 W and 5 s are selected as the optimal sound power and action time.

**Additional file 6: Figure S6.** In vitro ultrasound imaging performance. Fifteen grams of agarose and 500 mL of deionized (DI) water were mixed at a concentration of 3%. The mixture was repeatedly heated until the agarose dissolved. Then, after cooling, the agar gel was prepared as an in vitro ultrasound imaging model. The N_2_O-mbs (200 μL, 1 × 10^5^ bubbles/mL) PBS and C_3_F_8_-mbs (200 μL, 1 × 10^5^ bubbles/mL) PBS solutions were added to the gel model. Ultrasound imaging of the microbubbles was performed on an ultrasound system (Esaote, Italy). Finally, the echo intensities of regions of interest (ROI) were were quantitatively measured by ultrasound imaging software. The in vitro N_2_O-mb and C_3_F_8_-mb ultrasound imaging performance analysis is shown in Figure S6. The echo intensity of the B-mode ultrasound image within the ROI was calculated by ultrasound analysis software. The target sound intensity between the N_2_O-mbs group and the C_3_F_8_-mbs group was not significantly different (*p* > 0.05).

## Data Availability

The conclusions made in this manuscript are based on the data which are all presented and shown in this paper.

## Abbreviations

N_2_O-mbs: N_2_O microbubbles
C_3_F_8_-mbs: C_3_F_8_ microbubbles
HIFU: high-intensity focused ultrasound
LIFU: low-intensity focused ultrasound
SOSG: singlet oxygen sensor green
SDT: sonodynamic therapy
DPPC: 1,2-dihexadecanoyl-rac-glycero-3-phosphocholine
DSPE-mPEG2000: 1,2-distearoyl-sn-glycero-3phosphoethanolamine-*N*-[methoxy(polyethylene glycol)-2000
DAPI: 4′,6-diamidino-2-phenylindole
DiI: 1,1′-dioctadecyl-3,3,3′,3′-tetramethylindocarbocyanine perchlorate
DCFH-DA: 2′,7′-dichlorofluorescin diacetate
DAF-FM: 3-amino,4-aminomethyl-2′,7′-difluorescein, diacetate
CAM: Calcein-AM
CCK-8: Cell Counting Kit-8
CLSM: confocal laser scanning microscopy
TEM: transmission electron microscope
DMEM: Dulbecco’s modified Eagle’s medium
FBS: fetal bovine serum
PBS: phosphate-buffered saline
DI: deionized water
ROI: regions of interest
ROS: reactive oxygen species
NO: nitric oxide
