# Supplementary figures and images for: The Study of Enhanced High-Intensity Focused Ultrasound Therapy by Sonodynamic N2O Microbubbles
Source: Nanoscale Res Lett. 2019 Dec 16;14:381. doi: 10.1186/s11671-019-3219-0 (PMC6915195; doi:10.1186/s11671-019-3219-0)

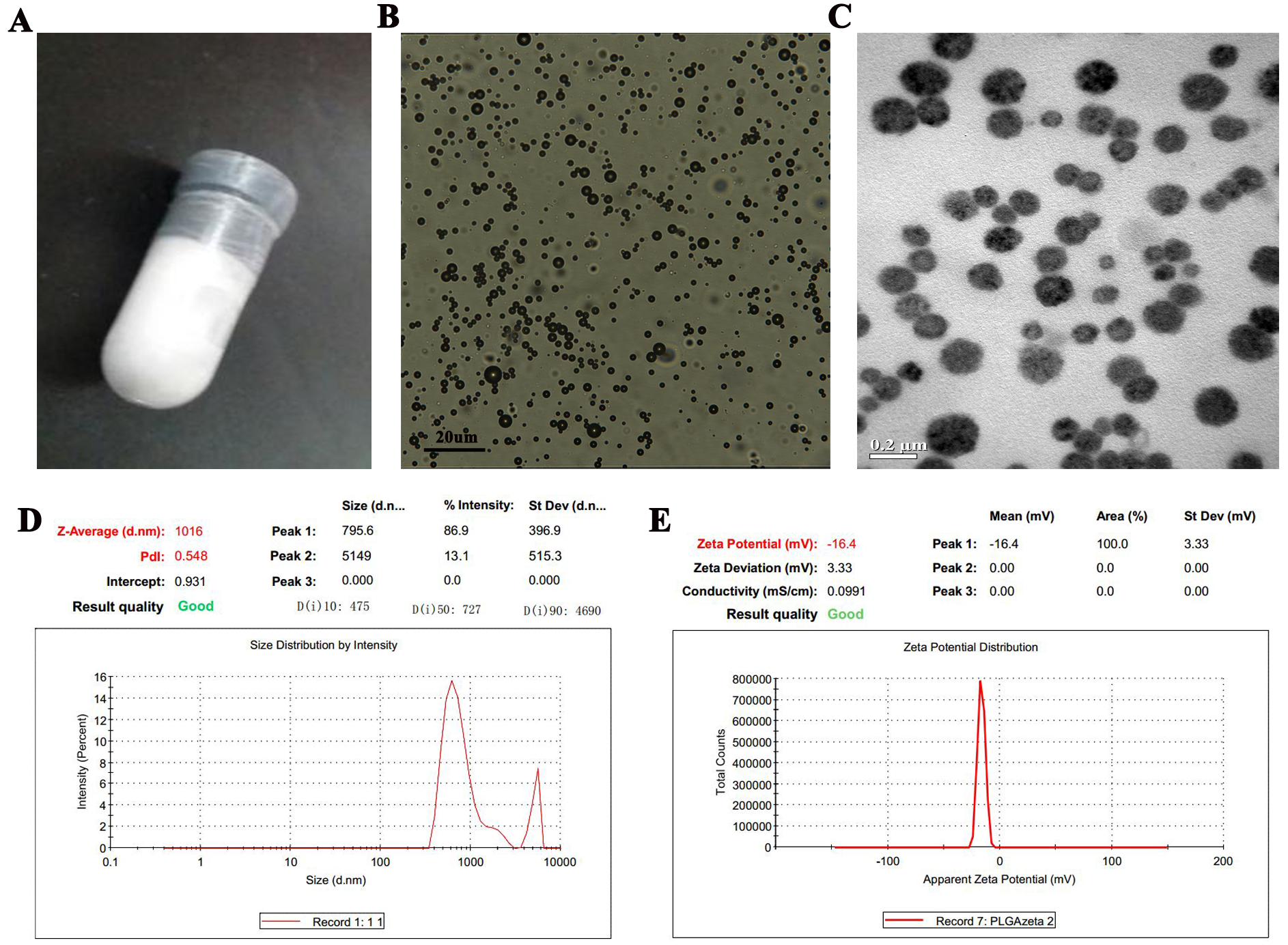

Supplement: Supplementary file 1 — Additional file 1: Figure S1. Basic representation of C3F8-mbs. A. Photographs of C3F8-mbs dispersed in deionized water; B. Image of C3F8-mbs under bright-field optical microscopy; C. Transmission electron microscope image of C3F8-mbs; D. Size distribution of C3F8-mbs; E. zeta potential of C3F8-mbs. [file 11671_2019_3219_MOESM1_ESM.tif]

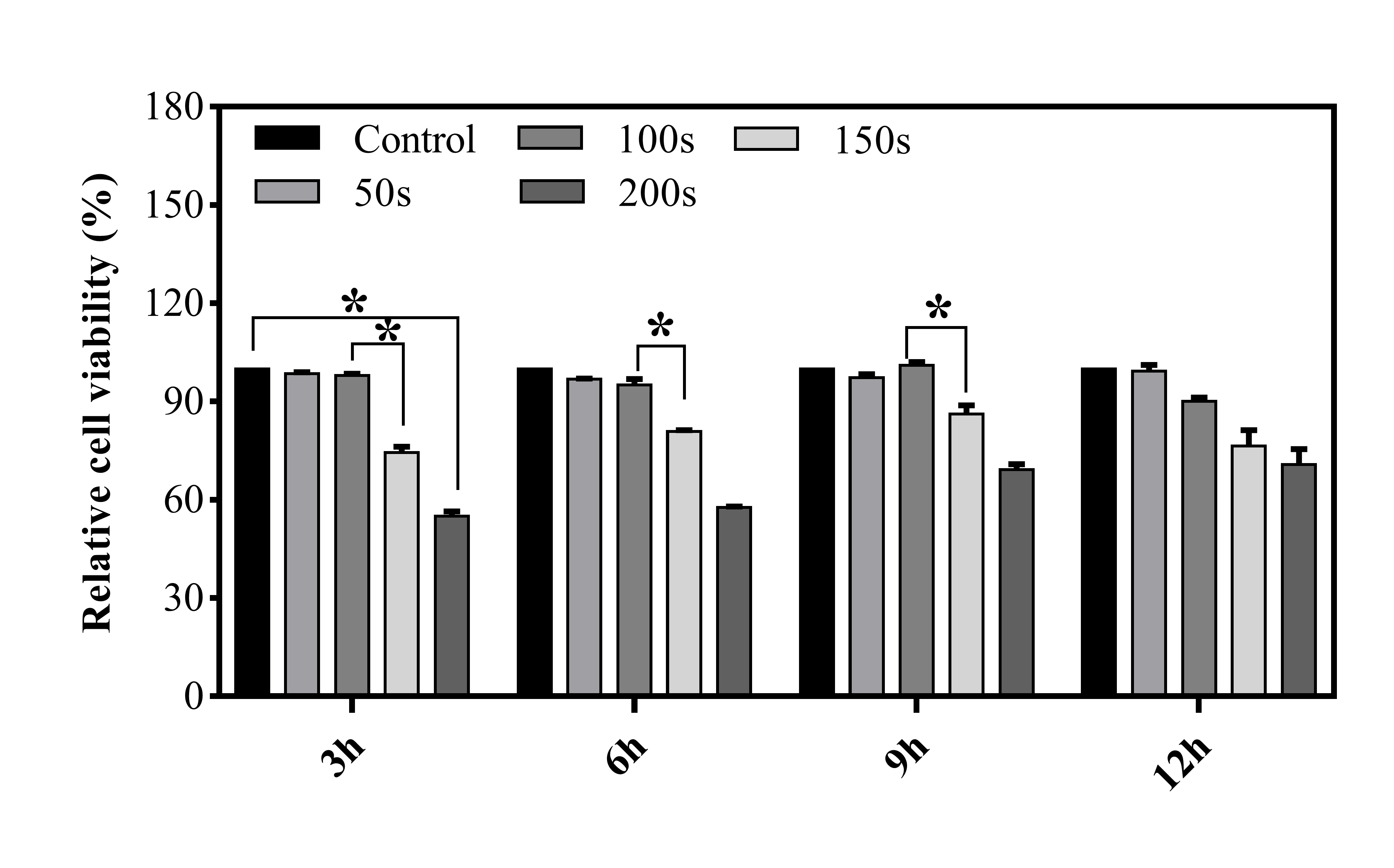

Supplement: Supplementary file 2 — Additional file 2: Figure S2. Determination of optimal LIFU irradiation time. (The data were shown as mean ± SD, n = 5 per group, *p < 0.05). To Select the optimal time for ultrasound, MDA-MB-231 cells were seeded in a 96-well plate at a density of 5000 cells per well for 24 h. And then use the strength of 2 W/cm2 ultrasonic processing cells, respectively for the 50 s, 100 s, 150 s and 200 s. After the indicated treatments, CCK8 solution (10 μL) was added in each well. Then, the MDA-MB-231 cells were cultured at 37 °C, 5% CO2 cell incubation box to save. At 3 h, 6 h, 9 h and 12 h, cell viabilities were determined by CCK-8 assay. The optical density of each well was measured at 450 nm with a Bio-Tek microplate reader. Cell viability of the control group was defined as 100%. The test results show (Figure S1) that the cells viability in 50 s and 100 s groups were more than 95% at 3 h, 6 h, 9 h and 12 h after treatment, and the difference in cell viability between the two groups is not statistically significant (p > 0.05). The cell viability decreased in 150 s and 200 s groups (p < 0.01), and the survival rate was 74% in 150 s group and 55% in 200 s group. Combined with the oxygen free radicals generated condition, 100 s was selected as the optimal time of ultrasound. [file 11671_2019_3219_MOESM2_ESM.tif]

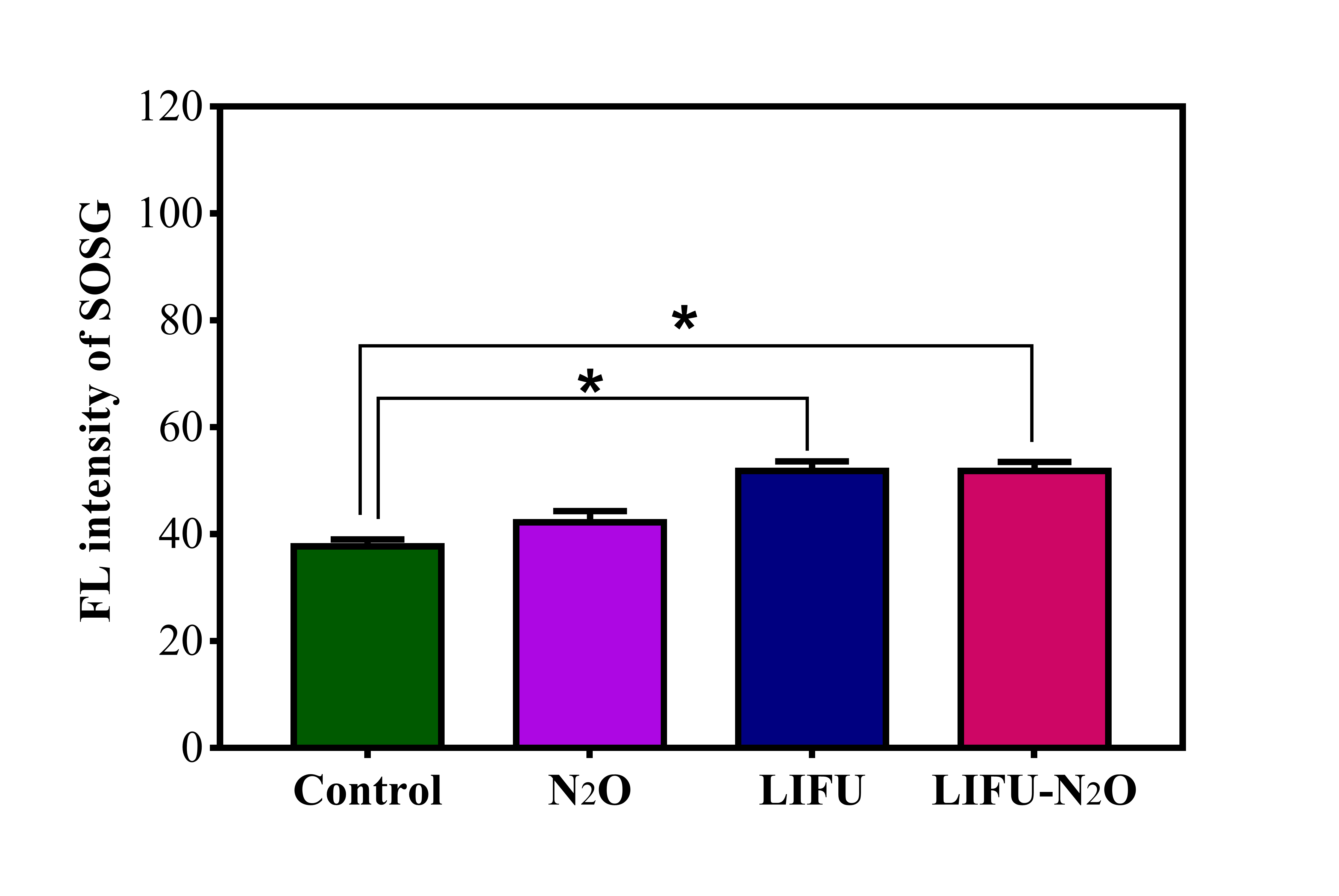

Supplement: Supplementary file 3 — Additional file 3: Figure S3. Quantitative analysis of 1O2 generation of N2O irradiated by LIFU. (The data were shown as mean ± SD, n = 5 per group, *p < 0.05). In aqueous solution, the difference between the N2O treated with ultrasound (LIFU-N2O group) and the N2O group was not statistically significant (p > 0.05). Compared with the control group, fluorescence intensity increased only in the LIFU group and LIFU-N2O group. And the addition of N2O did not increase the generation of oxygen free radicals in the LIFU-N2O group (p > 0.05). In the LIFU group and LIFU-N2O group, a small amount of oxygen free radicals was generated, possibly only because of the stimulation of ultrasound. [file 11671_2019_3219_MOESM3_ESM.tif]

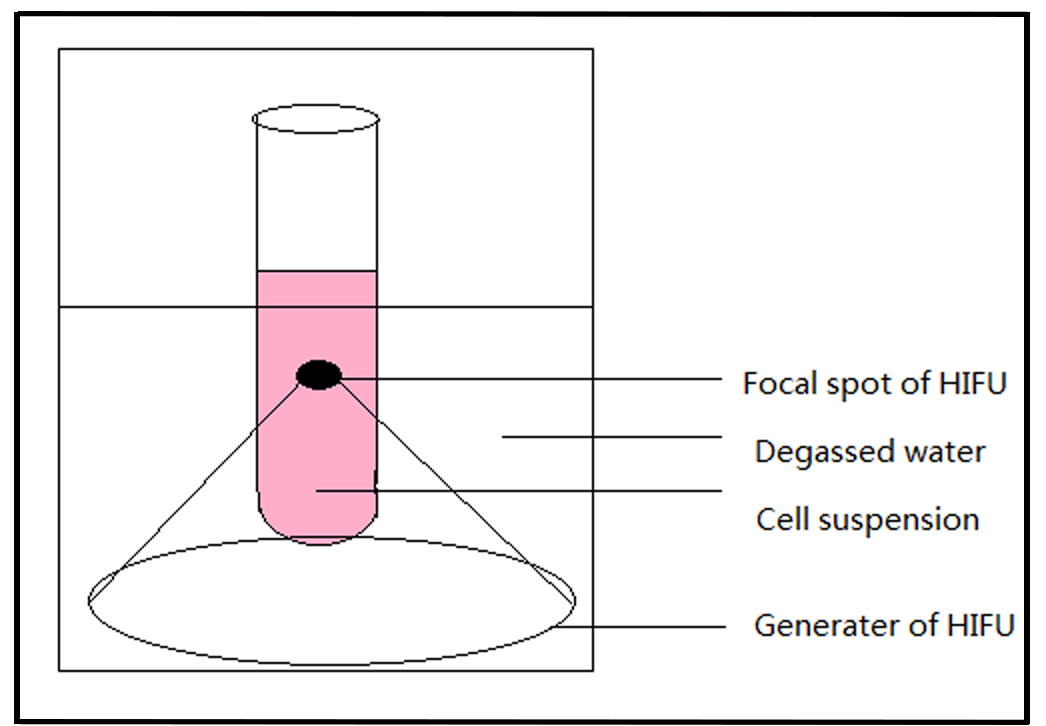

Supplement: Supplementary file 4 — Additional file 4: Figure S4. HIFU ablation cell suspension. [file 11671_2019_3219_MOESM4_ESM.tif]

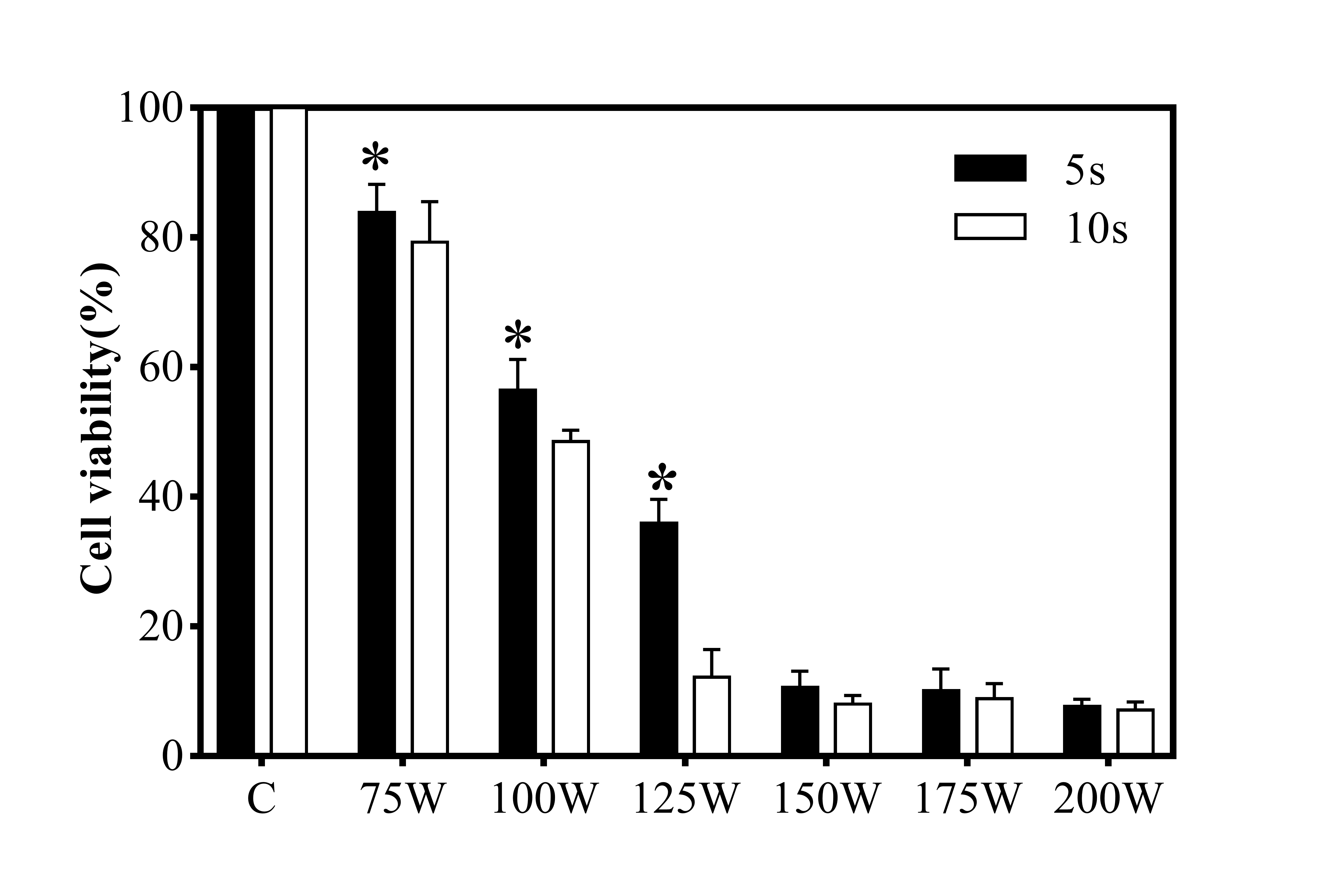

Supplement: Supplementary file 5 — Additional file 5: Figure S5. HIFU intensity and time selection. (The data were shown as mean ± SD, n = 5 per group, *p < 0.05). The prepared MDA-MB-231 cells suspension was grouped and treated with different acoustic power (75 W, 100 W, 125 W, 150 W, 175 W, 200 W) and different time (5 s, 10 s). Cell viability was detected with CCK8 assay. After the indicated treatments, MDA-MB-231 cells were inoculated in 96-well plates, with 5 wells in each group. CCK8 solution (10 μL) was added in each well and cultured at 37 °C for another 1 h. The optical density of each well was measured at 450 nm with a Bio-Tek microplate reader. Cell viability of the control group was defined as 100%. When the time was 5 s, the cell viability rate decreased significantly when the sound power was higher than 125 W (p < 0.05), and the cell survival rate no longer changed with the increase of the sound power (p > 0.05). At the same sound power and time of 10 s, the cell survival rate of each group did not change significantly. According to the experimental conditions, 125 W and 5 s are selected as the optimal sound power and action time. [file 11671_2019_3219_MOESM5_ESM.tif]

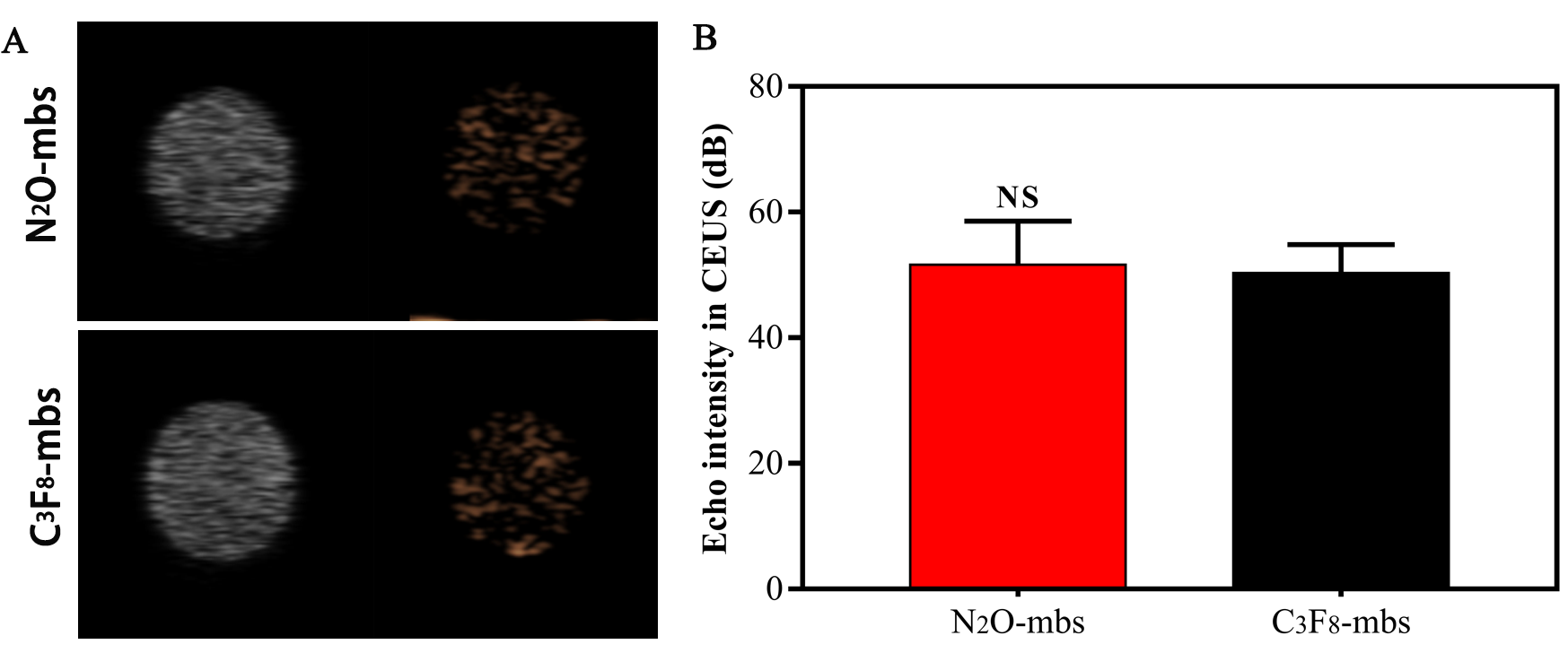

Supplement: Supplementary file 6 — Additional file 6: Figure S6. In vitro ultrasound imaging performance. Fifteen grams of agarose and 500 mL of deionized (DI) water were mixed at a concentration of 3%. The mixture was repeatedly heated until the agarose dissolved. Then, after cooling, the agar gel was prepared as an in vitro ultrasound imaging model. The N2O-mbs (200 μL, 1 × 10^5^ bubbles/mL) PBS and C3F8-mbs (200 μL, 1 × 10^5^ bubbles/mL) PBS solutions were added to the gel model. Ultrasound imaging of the microbubbles was performed on an ultrasound system (Esaote, Italy). Finally, the echo intensities of regions of interest (ROI) were were quantitatively measured by ultrasound imaging software. The in vitro N2O-mb and C3F8-mb ultrasound imaging performance analysis is shown in Figure S6. The echo intensity of the B-mode ultrasound image within the ROI was calculated by ultrasound analysis software. The target sound intensity between the N2O-mbs group and the C3F8-mbs group was not significantly different (p > 0.05). [file 11671_2019_3219_MOESM6_ESM.tif]
